# Cobalt and Titanium Alleviate the Methylglyoxal-Induced Oxidative Stress in *Pennisetum divisum* Seedlings under Saline Conditions

**DOI:** 10.3390/metabo13111162

**Published:** 2023-11-19

**Authors:** Bushra Ahmed Alhammad, Khansa Saleem, Muhammad Ahsan Asghar, Ali Raza, Abd Ullah, Taimoor Hassan Farooq, Jean W. H. Yong, Fei Xu, Mahmoud F. Seleiman, Aamir Riaz

**Affiliations:** 1 Biology Department, College of Science and Humanity Studies, Prince Sattam Bin Abdulaziz University, Al Kharj Box 292, Riyadh 11942, Saudi Arabia; 2 Department of Horticultural Sciences, The Islamia University of Bahawalpur, Bahawalpur 6300, Pakistan; 3 Department of Biological Resources, Agricultural Institute, Centre for Agricultural Research, ELKH, 2 Brunzvik St., 2462 Martonvásár, Hungary; 4 University of Chinese Academy of Sciences, Beijing 100049, China; 5 Bangor College China, A Joint Unit of Bangor University and Central South University of Forestry and Technology, Changsha 410004, China; 6 Department of Biosystems and Technology, Swedish University of Agricultural Sciences (SLU), 234 22 Lomma, Sweden; 7 Applied Biotechnology Center, Wuhan University of Bioengineering, Wuhan 430415, China; 8 Plant Production Department, College of Food and Agriculture Sciences, King Saud University, P.O. Box 2460, Riyadh 11451, Saudi Arabia; 9 Department of Crop Sciences, Faculty of Agriculture, Menoufia University, Shibin El-Kom 32514, Egypt

**Keywords:** microelements, stress protectant, methylglyoxal, glyoxalase pathway, plant diabetes, salinity stress

## Abstract

Salinity is considered to be a global problem and a severe danger to modern agriculture since it negatively impacts plants’ growth and development at both cellular- and whole-plant level. However, cobalt (Co) and titanium (Ti), multifunctional non-essential micro-elements, play a crucial role in improving plant growth and development under salinity stress. In the current study, Co and Ti impact on the morphological, biochemical, nutritional, and metabolic profile of *Pennisetum divisum* plants under three salinity levels which were assessed. Two concentrations of Co (Co-1; 15.0 mg/L and Co-2; 25.0 mg/L), and two concentrations of Ti (Ti-1; 50.0 mg/L and Ti-2; 100.0 mg/L) were applied as foliar application to the *P. divisum* plants under salinity (S1; 200 mM, S2; 500 mM, and S3; 1000 mM) stress. The results revealed that various morphological, biochemical, and metabolic processes were drastically impacted by the salinity-induced methylglyoxal (MG) stress. The excessive accumulation of salt ions, including Na^+^ (1.24- and 1.21-fold), and Cl^−^ (1.53- and 1.15-fold) in leaves and roots of *P. divisum*, resulted in the higher production of MG (2.77- and 2.95-fold) in leaves and roots under severe (1000 mM) salinity stress, respectively. However, Ti-treated leaves showed a significant reduction in ionic imbalance and MG concentrations, whereas considerable improvement was shown in K^+^ and Ca^2+^ under salinity stress, and Co treatment showed downregulation of MG content (26, 16, and 14%) and improved the antioxidant activity, such as a reduction in glutathione (GSH), oxidized glutathione (GSSG), Glutathione reductase (GR), Glyoxalase I (Gly I), and Glyoxalase II (Gly II) by up to 1.13-, 1.35-, 3.75-, 2.08-, and 1.68-fold under severe salinity stress in *P. divisum* roots. Furthermore, MG-induced stress negatively impacted the metabolic profile and antioxidants activity of *P. divisum*’s root and leaves; however, Co and Ti treatment considerably improved the biochemical processes and metabolic profile in both underground and aerial parts of the studied plants. Collectively, the results depicted that Co treatment showed significant results in roots and Ti treatment presented considerable changes in leaves of *P. divism* under salinity stress.

## 1. Introduction

Soil salinity, an important abiotic stressor across the globe, is negatively affecting crop productivity by impairing plant growth and development via osmotic, ionic, and/or nutritional imbalance in nutrition and plant metabolism. Furthermore, salt causes carbonyl-induced stress by generating highly reactive molecules such as methylglyoxal (MG) as a result of glycolysis [1]. Excessive salt-ion production, such as sodium (Na^+^), magnesium (Mg^2+^), potassium (K^+^), chloride (Cl^−^), carbonate (CO_3_^−2^), and calcium (Ca^2+^), which not only has a negative impact on plant growth and development (Bennett et al., 2012), but also causes nutritional imbalances like ammonium (NH_4_^+^) and nitrate ion (NO_3_^−^) [2]. The NH_4_^+^ imbalance resulted into subtle changes in glycolytic flux which resulted into over-accumulation of metabolic by-products such as methylglyoxal (MG) [3]. The MG is considered as one of the most toxic dicarbonyl metabolites, causing denaturation of phospholipids, nucleic acids, proteins, and amino acids such as arginine, lysine, and cysteine to form advanced-glycation end products (AGEs), which impair the functionality of their biological target cells in plants under abiotic stress [4]. Despite being highly reactive in nature, MG has harmful and/or signaling effects in plants. However, the signaling and/or harmful effects are entirely dependent on the MG content, plant species, and environmental stress [5,6,7]. Furthermore, MG also participates in the reduction of O_2_ which induces oxidative stress in plants [8]. However, plants also have well-defined two-step MG-detoxification mechanisms consisting of two metalloenzmes: Glyoxalase I (Gly I), and Glyoxalase II (Gly II). In this mechanism, Gly I and Gly II use GSH as a cofactor and convert the toxic MG into non-toxic D-lactate [9,10]. MG detoxification is one of the crucial factors in salinity stress tolerance, and the GSH seems to play a pivotal role in this regard. However, MG is a rather new concept in plant physiology and needs more scientific studies to better understand the MG-induced carbonyl stress in different plants and their effects on plant primary and secondary metabolism associated with the morphological alterations which resulted in yield and quality loss.

The exogenous trace elements’ application is considered to improve the salt tolerance in plants under abiotic stresses including salinity, drought, or heat stress [11,12]. Co and Ti are the emerging biostimulants and enhance stress-tolerance by reducing oxidative damage, improving the nutritional-quality and yield components of plants [12]. Previous studies revealed that a very low concentration of Co [13,14] and Ti [15,16] enhanced overall growth and development, nitrogen fixation, and improved quality and yield under abiotic stresses. Some previous studies also revealed that Co play a crucial role in nitrogen fixation in many legume and cereal crops and has a significant role in the synthesis of vitamin B12. Besides this, Co is a necessary component of key enzymes and proteins regulating the plant metabolism [17,18]. However, this concept is rather new in plant science and need more scientific knowledge to understand the full picture of their role in plant physiology and molecular biology. Grasses play an important role in land stabilization and rehabilitation and animal nutrition because of their high carbohydrates, fats, fiber, and protein content. *Pennisetum divisum*, is an important salt-sensitive grass species mainly used as fodder for desert animals such as camels [19,20]. Because of the prevailing salt conditions, it is necessary to develop a salt-tolerant strategy for *Pennisetum divisum*. Keeping in view the significance of *Pennisetum divisum* and its performance under saline conditions with possible mitigation impacts of Co and Ti, the current experiment was conducted with the following objectives: (i)To investigate the impact of salinity on the morphological, nutritional, biochemical, and metabolic profile of *P. divisum*, and how micronutrients (Co and Ti) could be effective to mitigate the hazardous effect of salinity. (ii) To explore the MG-induced carbonyl stress in grass tissues and its detoxifying pathway using Co and Ti as salinity-protectant micronutrients. (iii) To study the role of Co and Ti in mitigating the negative impact of saline stress by affecting the primary and secondary metabolism of studied grass species.

## 2. Materials and Methods

### 2.1. Planting Site, Soil Preparation, and Seedling Transplant

A pot experiment was conducted at the research area of the Department of Horticultural Sciences, The Islamia University of Bahawalpur. The plants were grown in a well-ventilated glasshouse at 30 °C in the day and 20 °C at night, with relative humidity of 15% in the day and 60% at night. A nursery of *P. divisum* was purchased from the local nursery in Bahawalpur at 2–4 true-leaf stage. Sand and garden soil in the ratio 1:1 was used as the planting medium. The pot sizes (selected to avoid root restriction in grass species) and properties of the initial experimental soil, including the initial status of nitrogen, phosphorus, and potassium, were evaluated, and the findings were reported earlier [21]. Based on the earlier studies, 5 kg of properly-sieved-soil filled plastic containers of 50 cm high and 20 cm wide. Seedlings of *P. divisum* were transplanted at 2–4 true-leaf stage into the soil-filled plastic bins. As with standard greenhouse-management methods, the potting mixture in each pot was fertilized with a complete nutrient solution of Hewitt’s nitrate (12 mM) nutrient solution [22].

### 2.2. Treatments

Based on our preliminary experimental results, there were 15 treatments in the current experiment consisting of three salinity levels, 200 mM (S1), 500 mM (S2), and 1000 mM (S3). Similarly, two levels of Co, i.e., 15.0 mg/L (Co-1) and 25.0 mg/L (Co-2), were in the form of cobalt sulphate (CoSO_4_), whereas two levels of Ti, i.e., 50.0 mg/L (Ti-1), and 100.0 mg/L (Ti-2), in the form of TiO_2_ were exogenously applied to the plants. Treatment arrangements are described in Table 1. For Co treatments, the Co-0 and, for Ti treatments, the Ti-0 were taken as control treatments. Saline conditions were provided in the soil in the form of irrigation water (NaCl) after two weeks from the day of transplant, while during the fourth week of transplant Co and Ti were applied to the transplanted seedlings in the form of foliar application twice a week for four consecutive weeks. During the 10th week, plants were harvested and the below-mentioned parameters were studied according to their standard protocol.

### 2.3. Parameters Studied

#### 2.3.1. Morphological Parameters

The morphological attributes, including leaf number/plant (LN/plant), were counted manually and means were recorded. Leaf area (LA) was recorded using a leaf area meter. Furthermore, after harvest, plant parts (roots and leaves) were carefully separated. Roots were thoroughly washed with distilled water and air dried. After that, shoot length (SL) and root length (RL) were recorded in “cm” using a measuring scale, while shoot fresh weight (SFW), and root fresh weight (RFW) were measured in “mg”, using a digital weighting balance. For dry biomass, plant samples were packed and labeled in paper bags and dried at 70 °C for 48 h in a hot dry oven. After the prescribed period of time, the following parameters for shoot dry weight (SDW), and root dry weight (RDW) and means were recorded, and were measured in “mg”.

#### 2.3.2. Nutrients Uptake

The sodium (Na^+^), chloride (Cl^−^), potassium (K^+^), and calcium (Ca^2+^) contents of dry leaves and root tissues were determined using a previously described method [23,24]. Briefly, the acid mixture (HNO_3_:HClO_4_; 5:1) was used to digest 0.1 g of ground, homogeneous dry plant material including leaves and roots. The Na^+^, Cl^−^, K^+^, and Ca^2+^ concentrations were measured from the digestion solution using the atomic absorption spectrometer (AA-7000, Shimadzu, Japan).

#### 2.3.3. Methylglyoxal Quantification Method

MG was quantified using the previously described protocol [8]. Briefly, 250 mg of fresh leaves and root samples were crushed using a mortar and pestle in the presence of liquid nitrogen. Later, 2.5 mL of HClO_4_ (0.5 M) was added to the mixture and was transferred to the micro-centrifuged tube and incubated on ice for 20 min. The extract was then centrifuged at 11,000× *g* for 10 min at 4 °C. The supernatant was moved to a new tube. In the next step, saturated potassium carbonate (K_2_CO_3_) was added gradually, and the pH was measured. With each K_2_CO_3_ addition, the supernatant was mixed thoroughly, and CO_2_ bubbles were allowed to come out. Then, the extract was kept at room temperature for 20 min and centrifuged at 11,000× *g* for 15 min. The supernatant was used for MG estimation. For MG estimation, a total volume of 1 mL is made up of 250 L of 1,2-diaminobenzene at 7.2 mM, 100 mL of HClO_4_ at 5 M, and 650 mL of neutralized supernatant. After 30 min of room-temperature incubation, the mixture was tested for absorbance at 336 nm. The amount of MG was calculated and represented as μmol/g FW using the standard curve MG (Sigma, St. Louis, MO, USA).

#### 2.3.4. Gly I and Gly II Quantification/MG-Detoxification Mechanism/Biochemical Assays

Gly I and Gly II activities in leaves and roots were examined to better understand the effect of Co and Ti treatment during salt stress on the MG-detoxification system. The determination of Gly I and Gly II was referred to the previous method [25], and their activities were calculated using the molar absorption coefficient of 3.37 × 10^3^ M^−1^ cm^−1^ (for S-D-lactoylglutathione) and 1.36 × 10^4^ M^−1^ cm^−1^ (2-nitro-5-thiobenzoic acid), respectively, and were expressed as nmol/g FW.

The glutathione reductase (GR) was determined as described earlier [26] by monitoring the GSH-dependent oxidation of NADPH. The GR activity was calculated using an extinction coefficient of 6.2/(mM cm). One unit of enzyme was the amount necessary to decompose 1.0 μmol of NADPH per min at 25 °C.

The contents of reduced glutathione (GSH) and that oxidized (GSSG) were estimated following the earlier method [27]. Fresh leaves and roots (0.5 g) were homogenized in 2.0 mL of 5% sulfosalicylic acid under a low temperature. The homogenate was centrifuged at 10,000 rpm for 10 min. To 0.5 mL of the supernatant, 0.6 mL of K-phosphate buffer (100 mM, pH 7.0) and 40 μL of 5′,5′-dithiobis-2-nitrobenzoic acid (DTNB) were added. After 2 min, the absorbance was recorded at 412 nm on a spectrophotometer (AA-7000, Shimadzu, Japan). GSSG was assayed using the same method in the presence of 2-vinylpyridine, and the GSH concentration was calculated from the difference between total glutathione and GSSG.

#### 2.3.5. Metabolites Extraction

All metabolites such as sugars and sugar alcohols (sucrose, maltose, rhamnose, fructose, fructose-6-P, fructose-1,6-BP, glucose, glucose-6-P, myo-inositol-P, myo-inositol, inositol, pinitol, ribulose-5-P, ribose, xylose, melibiose, trehalose, and mannose), amino acids (histidine, GABA, adenosine, adenine, serine, glycine-betaine, 5-methylcysteine, cysteine, leucine, valine, propanamine, butylamine, phenylalanine, lysine, proline, asparagine, glutamine, threonine, isoleucine, orinithine, omithine, ethanolamine, arginine, and 4-hydroxyproline), and organic acids (lactate, butyrate, 2-hydroxyyglutamate, 3-P-Glyceric acid, phosphoenolpyruvic acid, pyruvate, glyceric acid, 2-momo-isoutyrine, propanoic acid, glucuronate, gluconate, citrate, cis-aconitate, α-ketoglutarate, 2-hydroxyglutarate, 2-hydroxyglutarate, glutamate, malate, succinate, fumarate, maleate, oxaloacetate, aspartate, aspartic acid, and γ-aminobutyrate in roots, and leaves, of *P. divisum)* were studied using the earlier-documented procedure [28]. Sample volumes of 1 μL were analyzed with a Trace gas chromatograph coupled with a PolarisQ ion-trap mass spectrometer (GC-MS) equipped with an AS2000 auto sampler (Thermo Electron, Dreieich, Germany). Derivatized metabolites were evaporated at 250 °C in the splitless mode and separated on a 30 m × 0.25 mm RTX-5MS capillary column with a 0.25 mm coating equipped with an integrated 10 m guard column (Restak, Bad Homburg, Germany). Helium carrier gas flow was adjusted to 1 mL/m. The interface temperature was set to 250 °C and the ion source temperature to 220 °C. The oven temperature was kept constant for 3 min at 80 °C after each analysis. Mass spectra were recorded at 1 scan/s with a scanning range of 50 to 750 *m*/*z*. Metabolites were identified using comparison to pure standard (Sigma-Aldrich). In addition, the freely available Golm Metabolome Database [29] was of particular help in identifying several metabolites. All identified compounds matched the references using mass spectral data and chromatographic retention time. Relative levels of selected metabolites were determined automatically by integrating the peak areas of selective ions [30] with the processing setup implemented in Xcalibur 1.4 software (Thermo Electron, Dreieich, Germany). Relative-response ratio was calculated by normalizing the respective peak areas to the peak area of the internal standard ribitol and dividing the value by the dry weight of the sample. Measurements were performed in technical duplicates for each of the three replicates of control and the Co and Ti treated plants.

### 2.4. Statistical Analysis

A pot experiment was conducted with 15 treatments in total (three replications each). All the data were analyzed statistically using the analyses of variance technique (ANOVA) under a complete randomized design (CRD). Treatment means were analyzed statistically using the least significant difference (LSD) test at a 5% level of probability by using SPSS software, and the co-relation was found using OriginPro2021 (version 2022.01). The TBtools software was used to develop the heatmaps.

## 3. Results

### 3.1. Growth Indices

The results revealed that salinity stress negatively impacted the morphological characteristics of *Pennisetum divisum*. All the studied parameters including LN/plant, LA, RFW, RDW, RL, SFW, SDW, and SL decreased by 2, 7, 14, 18, 14, 8, 9, and 5% under 200 mM (low salt stress); 15, 23, 32, 37, 26, 19, 23, and 13% at 500 mM (moderate); and 26, 31, 45, 47, 35, 32, 37, and 18% under 1000 mM (severe) salinity stress, compared to the control treatment, respectively (Table 2). However, Co-1 application significantly improved the growth parameters under all salinity levels. Furthermore, Co-2 application reduced LN/plant by 3, 5, and 4% under low, moderate, and severe salt stress, respectively. While RDW and SDW decreased by 3 and 5% under S1 stress, SDW further decreased by 2% under 1000 mM salt stress compared to the control treatment, respectively. Similarly, Ti-2-treated plants showed an increment of 1.11-, 1.07-, 1.06-, 1.05-, 1.1-, 1.05-, 1.08-, and 1.03-fold under low (200 mM salinity) stress; 1.16-, 1.12-, 1.09-, 1.14-, 1.04-, 1.04-, 1.07-, 1.05-fold under moderate (500 mM salinity) stress; and 1.21-, 1.17-, 1.23-, 1.19-, 1.22-, 1.21-, 1.08-, 1.07-fold under severe (1000 mM salinity) stress in all the studied parameters, compared to the untreated plants, respectively (Table 2).

### 3.2. Nutritional Imbalance/Ionic Toxicity

**Figure 1:**
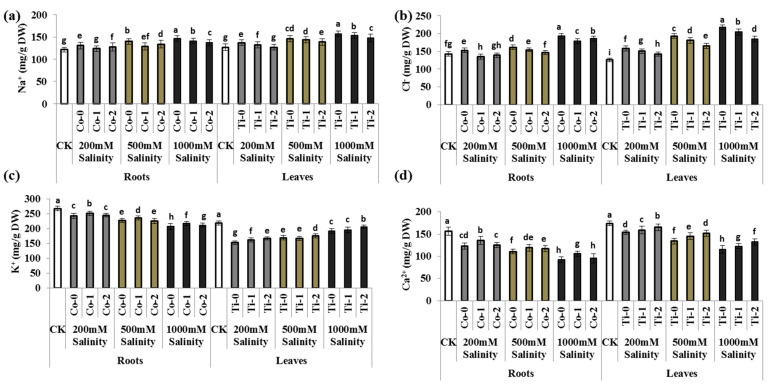
Exogenous application of cobalt and titanium’s effect on ionic toxicity and nutrient uptake of *Pennisetum divisum* under salinity stress. (**a**) Na^+^ (sodium), (**b**) Cl^−^ (chloride), (**c**) K^+^ (potassium), (**d**) Ca^2+^ (calcium ion), CK (control treatment), Co-0 (0 mg/L), Co-1 (15 mg/L), Co-2 (25 mg/L), Ti-0 (0 mg/L), Ti-1 (50 mg/L), and Ti-2 (100 mg/L). The different letters above columns are significantly different from each other at *p* ≤ 0.05 levels in each parameter (ANOVA) followed by the least significant difference test.

Nevertheless, salinity stress caused ionic toxicity by increasing Na^+^ and Cl^−^ ions and reducing Ca^2+^ and K^+^ in both roots and leaves of the *P. divisum*. Briefly, salinity stress enhanced the Na^+^ and Cl^−^ ions up to 1.08- and 1.26-fold in leaves and 1.07- and 1.06-fold in roots under 200 mM stress; 1.15- and 1.53-fold in leaves and 1.15- and 1.13-fold in roots under 500 mM salinity; and 1.24- and 1.73-fold in leaves and 1.21- and 1.35-fold in roots under 1000 mM salt stress in *P. divisum*, compared to the control treatment, respectively. Conversely, all the salinity levels significantly inhibited the K^+^ and Ca^2+^ ions (Figure 1). However, the Ti-1 and Co-1 treatments inhibited the Na^+^ and Cl^−^ ions under all saline stress levels, while K^+^ and Ca^2+^ ions were considerably improved in leaves and roots of *P. divisum* plants. However, Ti-2 treatment showed no significant changes in Na^+^, Cl^−^, K^+^, and Ca^2+^ under 1000 mM stress (Figure 1).

### 3.3. MG and Its Scavenging Enzymes

**Figure 2:**
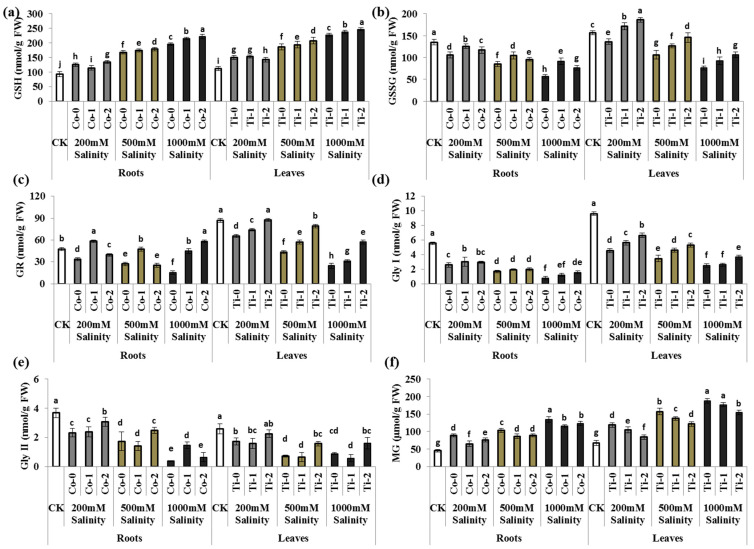
Exogenous application of cobalt and titanium’s effect on MG-induced oxidative stress, and its scavenging enzymes of *Pennisetum divisum* under salinity stress. (**a**) Reduced glutathione (GSH), (**b**) oxidized glutathione (GSSG), (**c**) glutathione reducase (GR), (**d**) glyoxalase I (Gly I), (**e**) glyoxalase II (Gly II), (**f**) methylglyoxal (MG), CK (control treatment), Co-0 (0 mg/L), Co-1 (15 mg/L), Co-2 (25 mg/L), Ti-0 (0 mg/L), Ti-1 (50 mg/L), and Ti-2 (100 mg/L). The different letters above columns are significantly different from each other at *p* ≤ 0.05 levels in each parameter (ANOVA) followed by the least significant difference test.

Salinity stress exerted MG-induced oxidative stress in *P. divisum* plants. MG showed an up-regulation of 1.75-, 2.32, and 2.77-fold in *P. divisum’s* leaves, whereas roots showed an increment of 1.96-, 2.27-, and 2.95-fold under 200 mM, 500 mM, and 1000 mM salt stress, compared to the control treatment, respectively. In contrast, the Ti-2 application significantly reduced the MG content in leaves by 45 and 22% under 200 mM and 500 mM stress, respectively. However, no significant change was observed under sever salinity stress (1000 mM). In addition, in *P. divisum* roots, Co-1 application downregulated MG content by 26, 16, and 14%, irrespective of the salinity stress compared to untreated *P. divisum* roots, respectively (Figure 2f).

In response to oxidative stress, for the antioxidants such as GSH, activity was enhanced by 1.32-, 1.65-, 2.01-fold in leaves and 1.35-, 1.80-, 2.11-fold in *P. divisum* roots under 200 mM, 500 mM, and 1000 mM salt stress, compared to the control treatment, respectively (Figure 2a). However, leave’s antioxidants, i.e., GSSG, GR, Gly I, and Gly II, showed an inhibition of 13, 25, 52, and 33% under 200 mM stress; 32, 50, 63, and 72% under 500 mM stress; and 51, 71, 73, and 65% under 1000 mM salt stress compared to the control, respectively. Similar to leaves, the roots undergo a significant reduction in antioxidants (GSSG, GR, Gly I, and Gly II) by 21, 29, 53, and 36% under 200 mM stress; 37, 42, 69, and 52% under 500 mM salt stress; and 58, 67, 86, and 89% under 1000 mM salinity stress compared to the control treatment, respectively (Figure 2b–e). The Ti-2-treated plants further improved by 1.37-, 1.34-, 1.45-, and 1.31-fold, whereas GSH reduced by 4% under 200 mM stress compared to untreated leaves, respectively. Further, Ti-2-treated leaves enhanced all the studied antioxidants including GSH, GSSG, GR, Gly I, and Gly II up to 1.12-, 1.37-, 1.83-, 1.53-, and 2.21-fold under 500 mM stress, and 1.08-, 1.39-, 2.31-, 1.46-, 1.79-fold under 1000 mM salt stress compared to the untreated plant. Similar results were observed in Co-treated leaves, respectively. Similarly, the Co-2-treated roots showed an increment of all the studied antioxidants (GSH, GSSG, GR, Gly I, and Gly II) of up to 1.07-, 1.12-, 1.17-, 1.14-, and 1.32-fold under 200 mM stress, whereas GSH, GSSG, Gly I, and Gly II 1.06-, 1.12-, 1.18-, and 1.43-fold. Meanwhile, GR reduced by 6% under moderate stress, and 1.13-, 1.35-, 3.75-, 2.08-, and 1.68-fold under 1000 mM salt stress compared to untreated plants, respectively (Figure 2). A similar pattern was observed in Ti-treated roots.

### 3.4. Metabolic Response

**Figure 3:**
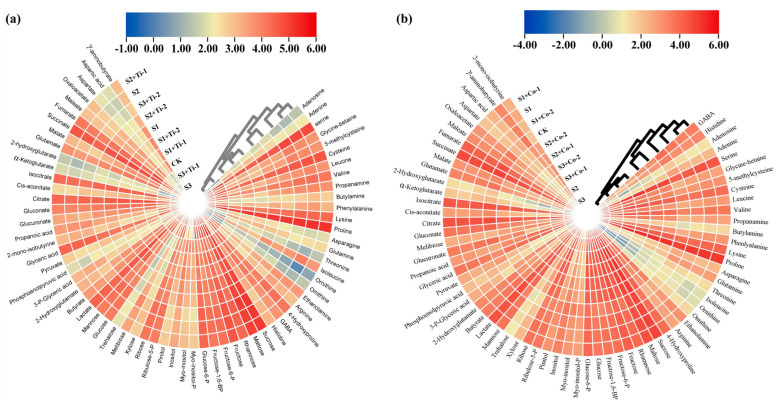
Exogenous application of cobalt and titanium effect on the metabolic profile of *Pennisetum divisum’s* (**a**) leaves, and (**b**) roots under salinity stress. CK (control treatment), S1 (salinity 200 mM), S2 (Salinity 500 mM), S3 (Salinity 1000 mM), Co-1 (15 mg/L), Co-2 (25 mg/L), Ti-0 (0 mg/L), Ti-1 (50 mg/L), and Ti-2 (100 mg/L). The heatmap was developed using TBtools software using log-2 values. Each colored cell on the map corresponds to a normalized log-response value of the studied metabolic activity, with samples in columns and metabolites in rows. Red color represents the highest metabolic level, whereas blue color indicates the lowest metabolic levels.

The metabolic profiles of the roots and leaves of *P. divisum* were modified with the interactive effect of the salt, Co, and Ti treatments. Briefly, *P. divisum* roots showed an up-regulation of sugar and sugar alcohols including sucrose, maltose, rhamnose, fructose, fructose-6-P, fructose-1,6-BP, glucose, glucose-6-P, ribulose-5-P, ribose, xylose, melibiose, trehalose, and mannose compared to the control treatment, respectively (Figure 3b).

However, myo-inositol-P, myo-inositol, inositol, and pinitol were downregulated under all stress levels, compared to control treatment, respectively. Furthermore, Co-2-treated roots presented a reduction in sugars and alcohols (sucrose, maltose, rhamnose, fructose, fructose-6-P, fructose-1,6-BP, glucose, glucose-6-P, ribulose-5-P, ribose, xylose, melibiose, trehalose, and mannose) under all stress levels when compared to the untreated, respectively. Although, the sugar alcohols including myo-inositol-P, myo-inositol, inositol, and pinitol showed an up-regulation (Figure 3b). The *P. divisum* leaves also showed the same trend under salinity, Co, and Ti treatments (Figure 3a).

The organic acids, such as lactate, butyrate, 2-hydroxyyglutamate, phosphoenolpyruvic acid, pyruvate, glucuronate, gluconate, citrate, cis-aconitate, α-ketoglutarate, 2-hydroxyglutarate, 2-hydroxyglutarate, glutamate, malate, succinate, fumarate, maleate, oxaloacetate, aspartate, aspartic acid, and γ-aminobutyrate, were considerably downregulated under all saline stress levels compared to the control treatment in *P. divisum* leaves. However, 3-P-Glyceric acid, glyceric acid, 2-momo-isoutyrine, and propanoic acid considerably improved up to and under 500 mM and 1000 mM of stress compared to the control, respectively (Figure 3a).

However, Ti-2-treated leaf organic acids, i.e., lactate, butyrate, 2-hydroxyyglutamate, phosphoenolpyruvic acid, pyruvate, glucuronate, gluconate, citrate, cis-aconitate, α-ketoglutarate, 2-hydroxyglutarate, 2-hydroxyglutarate, glutamate, malate, succinate, fumarate, maleate, oxaloacetate, aspartate, aspartic acid, and γ-aminobutyrate, showed an increment when compared to the untreated plants, respectively. However, 3-P-Glyceric acid, glyceric acid, 2-momo-isoutyrine, and propanoic acid were significantly reduced under Ti-2-treated leaves. The same trend was shown by the roots under all the applied treatments with few exceptions (Figure 3a).

The leaf amino acids’, including serine, glycine-betaine, 5-methylcysteine, cysteine, leucine, valine, propanamine, butylamine, phenylalanine, lysine, proline, isoleucine, ethanolamine, arginine, and 4-hydroxyproline, contents were enhanced irrespective of the salinity stress, compared to control treatment, respectively. Conversely, the GABA, histidine, adenosine, adenine, asparagine, glutamine, threonine, orinithine, and omithine showed a reduction under all the investigated saline levels (Figure 3a).

As compared to control leaves, the Ti-2-treated leaves suppressed the serine, glycine, 5-methylcysteine, cysteine, leucine, valine, propanamine, butylamine, phenylalanine, lysine, proline, isoleucine, ethanolamine, arginine, and 4-hydroxyproline contents, while the levels of GABA, histidine, adenosine, adenine, asparagine, glutamine, threonine, orinithine, and omithine were increased (Figure 3a). In addition, the roots showed the same pattern of reduction under salinity, and Co-2 application significantly improved the amino acid levels in *P. divisum* (Figure 3b).

## 4. Discussion

Over-salted soils are one of the most threatening abiotic factors negatively impacting plant growth and development at both cellular- and whole-plant level, which is threating the sustainability of agriculture and ultimately the environment. Salt stress reduces crop yield and quality in many ways including through ionic and osmotic imbalance, metabolic changes, nutritional deficiency, and impaired physiological and biochemical growth [1]. Furthermore, over-accumulation of salt ions such as Na^+^ and Cl^−^ resulted in the ionic imbalance which causes necrosis in leaves and resulted in decreased leaf area, net productivity, and crop yield. Another study suggested that roots are more susceptible to salinity compared to shoots [31,32,33]; contrary to these results, the current study revealed that roots and aerial parts of *P. divisum* both were hindered under salinity stress. The current results indicated that higher accumulation of salt ions resulted not only in reduced morphological growth but also affected Ca^2+^ and K^+^ in *P. divisum* roots and leaves; some previous studies were found in line with these results [34,35,36]. Moreover, another potential salinity-induced ion-toxicity mechanism is that the excessive Na^+^ replaces K^+^, affecting many physiological, biochemical and metabolic attributes in stressed plants; these findings correlate with the current results [37,38]. The results also revealed that Na^+^/K^+^ imbalance may cause Ca^2+^ deficiency in both roots and shoots of plants subjected to salt stress which resulted in nutritional scarcity and/or imbalance. Studies also revealed that oxidative stress also induces Ca^2+^ deficiency in salinity-stressed plants [38,39]; these studies were found to be parallel to the current findings. Moreover, exogenous Co and Ti application considerably improved the morphological attributes, reduced the ionic toxicity, improved the nutritional status, and enhanced the abiotic stress tolerance of different plants [13,15,16,40,41,42]; these findings were found to be indirectly in line with our results. Another study on rice under salinity stress was found to be indirectly parallel with our results, where silicon considerably enhanced the rice growth and development under over-salted conditions [43]. However, to the best of our knowledge, no studies were found describing Co and Ti transport mechanisms in plants, including inter and intracellular ionic fluxes, and Ti and Co form a relationship with other nutrients. To better understand this phenomenon more studies covering electrophysiological aspects are needed in the future.

Osmolytes, such as amino acids and sugars, play a significant role in salt-stress tolerance in plants. The excessive production of amino acids especially proline and glycine-betaine are indirectly involved in the scavenging of oxidative stress by activating many enzymatic reactions in plants under salt stress. Furthermore, amino acids play a crucial role in osmotic-stress tolerance in plants under abiotic stress [44,45]. These results were found to be in line with the current results. Co application considerably reduced the salinity stress by improving and/or altering metabolic profile of *P. divisum* under salinity stress; these results were found to be indirectly in line with the previous findings [15,25,40,46]. Moreover, some recent studies suggested that exogenous microelement application, including Ti, considerably improved plant growth and development by improving the metabolic profile, including through amino acids in plants subjected to different abiotic stresses [47,48,49,50]; these results were found to be in accordance with our outcomes. Although, no evidence was found on Co and Ti’s role in inducing metabolic response in the salt-stressed *P. divisum*. Additionally, the current study opens doors for future research into the role of Co and Ti activating and/or altering metabolic response, and the biological mechanisms involved in plants subjected to abiotic stress.

**Figure 4:**
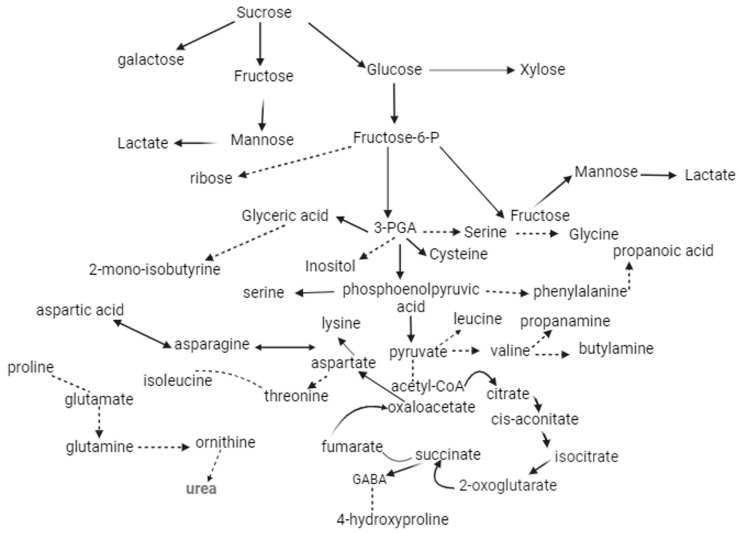
Schematic diagram of metabolites involved in *P. divisum’s* tissues under salinity stress.

Similar to amino acids, sugars also play a crucial role in salt-stress tolerance by maintaining carbon metabolism, osmotic adjustments, and membrane stabilization in different plants species. Another study suggested that higher Cl^−^ content resulted in increased sugar concentration in plant tissues [51,52,53]; these studies corroborate our results. Besides the beneficial effects, sugars also act as a “double-edge sword” in plants under abiotic stress. A schematic diagram of proposed metabolic activity including organic acids, amino acids, sugars, and sugar alcohols (Figure 4). The ionic imbalance leads to nitrogen-containing-nutrient deficiency which ultimately disturbs the carbon metabolism resulting in the over-production of MG, and highly reactive dicarbonyl species induce oxidative stress in plants under abiotic stress [54,55,56]. However, this is rather a new concept in plant physiology and needs more scientific research to understand the complete mechanism in plant biology and/or molecular biology. With limitations to N-containing nutrients the over-accumulation of Cl^−^ and excessive production of MG indicates the complex role of sugars in salt-sensitive *P. divisum* plants. Some previous studies on different plants, such as *Hordeum vulagre* L. [57], *Solanum lcopersicum* [46], *Triticum aestivum* L. [58], *Corchorus olitorius* [59], and *Leptocohloa fusca* [60], studied the MG-induced oxidative stress and its detoxification mechanisms under salinity and drought stress, respectively. However, to the best of our knowledge no studies were found on the exogenous Co and Ti application-alleviating MG-induced oxidative stress in plants. Although, few studies on exogenous osmolytes alleviating oxidative stress in plants subjected to salinity stress were found to be indirectly in line with the current results [25,59]. In future, more studies are needed to understand the Ti and Co biological pathways involved in mitigating the hazardous effects of MG-induced oxidative stress in plants.

Plants combat MG-induced oxidative stress by activating their natural defense mechanism consisting of two metallo-enzymes, including Glyoxalase I (Gly I) and Glyoxalase II (Gly II) and uses GSH as a co-factor. With limitations to the genetic study involved in activating Gly I and Gly II, the current results were found in accordance with the previous findings describing the MG-detoxification mechanism under high temperature, salinity, and ammonium stresses in different plant species [8,59,61,62]. These enzymes detoxify toxic MG into lactate/lactic acid, which later convert into non-toxic pyruvate (the end product of the Glyoxalase pathway) [5,54,61].

Similar to amino acids, organic acids such as citrate, pyruvate, maleate, succinate, and so on are considered to be important bio-stimulants in plants that are under abiotic stress. Some previous studies revealed that citric acid application significantly improved the overall growth and yield of different plant species by improving the antioxidant’s activity, enhancing primary and secondary metabolites, reducing osmotic stress, and enhancing the sugar content by activating other metabolites involved in the tricarboxylic acid cycle (TCA) [63,64,65,66]. These studies were found to be indirectly parallel with the current findings. Although, it is still unclear how Co and Ti influence the plant’s physiological processes and the metabolic profile of different plants under abiotic stress. A plethora of knowledge is already available on salinity affecting plants, including grass species growth and development [19,20,67]; however, microelements including Co and Ti alleviate MG-induced oxidative stress by altering the metabolic response in plants, the research for which is still in its rudimentary stage and needs further studies in the future.

## 5. Conclusions

To conclude, over-salted soils negatively impact the *P. divisum’s* growth. Briefly, the salinity stress induced ionic stress in *P. divisum* tissues (roots and leaves) and resulted in a higher accumulation of salt ions, including Na^+^ and Cl^−^, which resulted in reduced morphological growth. Furthermore, the excessive salt ions altered the Ca^2+^ and K^+^ concentrations, which significantly affected sugars/carbohydrates metabolism in plants and ultimately increased the MG content in both leaves and roots of the *P. divisum*. The excessive sugar accumulation induced “diabetes-like symptoms” in *P. divisum* plants and negatively affected the plant’s primary and secondary metabolites, including amino and organic acids. Further, the over-accumulation of MG initiated the MG-detoxification mechanism, which consisted of two enzymes named Gly I and Gly II along with other antioxidants such as GSH. Gly I and II reduced the toxic MG into lactate using GSH as a co-factor, and later it was converted into non-toxic pyruvate. The higher production of organic acids including lactate, pyruvate, etc., is the indication that organic acids are also involved in MG-induced oxidative stress in plants. Conversely, the exogenous Co and Ti application considerably improved the morphological growth, nutritional status, and metabolic profile of *P. divisum* by reducing ionic and MG-induced oxidative stress in *P. divisum* plants. Furthermore, the current results revealed that Co-2 application showed significant changes in *P. divisum’s* roots, while Ti-2 application enhanced the growth in the aerial parts of the plants. However, the full picture of biological pathway/s involved in MG detoxification using microelements (Ti and Co) as stress-protectants is still missing.

## Figures and Tables

**Table 1:** Details of the treatments used in the experiment.

SR	Treatment	Treatment Explanation
1.	T0	CK (control)
2.	T1	S1 (200 mM salt)
3.	T2	S1 + C0-1
4.	T3	S1 + Co-2
5.	T4	S1 + Ti-1
6.	T5	S1 + Ti-2
7.	T6	S2 (500 mM salt)
8.	T7	S2 + Co-1
9.	T8	S2 + Co-2
10.	T9	S2 + Ti-1
11.	T10	S2 + Ti-2
12.	T11	S3 (1000 mM salt)
13.	T12	S3 + Co-1
14.	T13	S3 + Co-2
15.	T14	S3 + Ti-1
16.	T15	S3 + Ti-2

Ti, Co, and S refer to titanium, cobalt and salt, respectively.

**Table 2:** Exogenous application of cobalt and titanium’s effect on morphological attributes of *Pennisetum divisum* under salinity stress.

	LN/Plant	LA (cm^2^)	RFW (mg)	RDW (mg)	RL (cm)	SFW (g)	SDW (mg)	SL (cm)
CK	14.6 b	25.71 ab	15.73 a	10.62 a	23.72 a	37.86 a	21.81 a	56.54 a
200 mM Salinity (S1)	14.2 bc	23.69 d	13.47 c	8.62 c	20.22 d	34.58 d	19.65 d	53.55 d
S1 + Co-1	15.57 a	24.65 c	15.66 a	9.18 b	21.85 b	35.65 c	20.6 c	54.03 d
S1 + Co-2	13.66 cd	23.62 d	14.27 b	8.34 c	20.69 d	34.86 d	18.3 e	53.96 d
S1 + Ti-1	15.3 a	25.78 a	13.65 c	8.29 c	21.25 c	35.82 c	20.73 c	54.73 c
S1 + Ti-2	15.71 a	25.28 b	14.22 b	9.07 b	22.26 b	36.41 b	21.27 b	55.54 b
500 mM Salinity (S2)	12.31 f	19.71 g	10.65 f	6.65 g	17.52 gh	30.33 h	16.65 g	48.72 h
S2 + Co-1	13.15 de	21.61 e	12.16 d	7.58 d	18.7 e	32.61 f	18.63 e	50.73 f
S2 + Co-2	11.58 g	20.72 f	11.37 e	7.15 ef	17.88 fg	31.7 g	15.6 h	49.71 g
S2 + Ti-1	13.71 c	21.77 e	10.64 f	6.83 fg	17.68 g	33.71 e	18.74 e	50.73 f
S2 + Ti-2	14.34 b	22.05 e	11.69 e	7.55 de	18.3 ef	31.59 g	17.78 f	51.39 e
1000 mM Salinity (S3)	10.75 h	17.55 i	8.57 j	5.58 i	15.3 j	25.37 l	13.62 j	46.19 j
S3 + Co-1	11.71 g	19.67 g	10.21 gh	6.71 g	17.11 hi	27.68 j	14.68 i	48.92 h
S3 + Co-2	10.23 h	17.98 i	9.36 i	6.21 h	16.84 i	26.05 k	13.34 j	47.62 i
S3 + Ti-1	11.63 g	18.56 h	9.83 h	6.08 h	16.71 i	28.86 i	15.79 h	48.54 h
S3 + Ti-2	12.99 e	20.54 f	10.5 fg	6.66 g	18.61 e	30.75 h	14.75 i	49.67 g

LN/plant (leaf number per plant), LA (leaf area), RFW (root fresh weight), RDW (root dry weight), RL (root length), SFW (shoot fresh weight), SDW (shoot dry weight), SL (shoot length), CK (control treatment), Co-1 (15 mg/L), Co-2 (25 mg/L), Ti-1 (50 mg/L), and Ti-2 (100 mg/L). The values marked with different letters are significantly different from each other at *p* ≤ 0.05 levels in each parameter (ANOVA) followed by the least significant difference test.

## Data Availability

Data is presented within this article.
